# Sandpipers go with the flow: Correlations between estuarine conditions and shorebird abundance at an important stopover on the Pacific Flyway

**DOI:** 10.1002/ece3.7240

**Published:** 2021-02-28

**Authors:** Rachel Canham, Scott A. Flemming, David D. Hope, Mark C. Drever

**Affiliations:** ^1^ Environment and Climate Change Canada Pacific Wildlife Research Centre Delta BC Canada; ^2^ Environment and Climate Change Canada Canadian Wildlife Service Ottawa ON Canada

**Keywords:** biofilm, Fraser River, freshet, migration, Pacific Dunlin, Salinity, Western Sandpiper

## Abstract

Estuaries of major rivers provide important stopover habitat for migratory birds throughout the world. These estuaries experience large amounts of freshwater inputs from spring runoff. Understanding how freshwater inputs affect food supply for migrating birds, and how birds respond to these changes will be essential for effective conservation of critical estuarine habitats. We estimated trends over time in counts of Western Sandpiper (*Calidris mauri*) and Pacific Dunlin (*Calidris alpina pacifica*) during northward migration on the Fraser River estuary, British Columbia, Canada, where shorebirds feed extensively on intertidal biofilm and invertebrates. We also examined whether counts were correlated with a suite of environmental variables related to local conditions (precipitation, temperature, wind speed and direction, solar radiation, tidal amplitude, and discharge rates from the Fraser River) during a total of 540 surveys from 1991 to 2019. Counts of Western Sandpiper declined ~54% (−2.0% per annum) over the entire study period, and 23% from 2009 to 2019 (−0.9% per annum). Counts of Pacific Dunlin did not show a statistically significant change over the study period. Counts of shorebirds were lower when discharge from the Fraser River was high, which we propose results from a complex interaction between the abrupt changes in salinity and the estuarine food web related to the quantity or quality of intertidal biofilm. Counts were also higher when tidal amplitude was lower (neap tides), potentially related to longer exposure times of the mudflats than during spring tides. Effects of wind are likely related to birds delaying departure from the stopover site during unfavorable wind conditions. The negative trend in migrating Western Sandpipers is consistent with declines in nonbreeding areas as observed in Christmas Bird Counts. Understanding causes of population change in migratory shorebirds highlights the need for research on mechanistic pathways in which freshwater inputs affect food resources at estuarine stopovers.

## Graphical abstract

Shorebird counts at a critical coastal stopover site have declined over a 29 year period (1991–2019). Counts were correlated with tidal amplitude, wind, and river discharge. We hypothesize a complex interaction between the abrupt changes in salinity from freshwater inputs, a decrease in quality and quantity of intertidal biofilm, and shorebird counts.

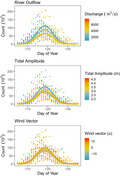

## 1 INTRODUCTION

Springtime is a period of rapid environmental change. Increasing photoperiods in spring stimulate plant growth, warm the atmosphere, and snowmelt redistributes freshwater from terrestrial to riparian and marine environments. The advent of spring co‐occurs with another natural phenomenon in the Northern Hemisphere: the northward migration of hundreds of thousands of shorebirds. During spring migration, many species fly thousands of kilometers while expending significant amounts of energy (Maillet & Weber, 2006; Guglielmo, 2010) and require safe stopover sites that are rich in food resources for refueling (Butler et al., 2001; Iverson et al., 1996; Warnock, 2010; Warnock & Bishop, 1998). The productive freshwater‐saltwater transition zones of estuaries along migration routes comprise large mud and sand flats with abundant prey and therefore act as critical stopover sites for a diversity of shorebirds (Butler et al., 2001; Harrington et al., 2002). However, many intertidal flats across the globe face significant human disturbance through construction of jetties and sea walls, “beach nourishment,” and development of intertidal habitat and are increasingly threatened by climate change and sea‐level rise (Galbraith et al., 2014; Murray et al., 2014, 2018).

Worldwide, monitoring programs report widespread declines of shorebird populations (Bart et al., 2007; Murray et al., 2018; Rosenberg et al., 2019; Smith et al., 2020; Wetlands International, 2012; Zöckler et al., 2013). Anthropogenic habitat alteration at nonbreeding and stopover sites is thought to be one critical driver of shorebird trends (Murray et al., 2014; Studds et al., 2017; Thomas et al., 2006). Loss or degradation of key migratory stopovers can have significant adverse effects on a species’ viability, which can lead to rapid population declines (Studds et al., 2017) and even possibly extinction (Weber et al., 1999). Anthropogenic disturbance and habitat loss can decrease available foraging habitat and degrade habitat quality at stopover sites, which may negatively impact body condition and thus shorebird survival (Studds et al., 2017). Additionally, annual variation in environmental conditions at stopover sites, such as food availability, weather, and risk of predation, may influence shorebird site usage during migration. Yet, the relationship between environmental conditions and shorebird requirements remains unknown at many stopover sites. Understanding what drives shorebird migration patterns and associations with environmental conditions at stopover sites could provide important information on stopover site resource availability and inform shorebird conservation.

Food supply is a key component of habitat quality and influences stopover site selection, migration timing, and body condition upon arrival at breeding grounds (Butler et al., 2001). Thus, food supply on migration is integral for shorebird survival and reproductive success (Butler et al., 2001). Recent findings indicate small‐bodied shorebirds consume large quantities of intertidal biofilm directly as a food source, or indirectly through consumption of invertebrates (e.g., mollusks, copepods, and small crustaceans) that feed on biofilm (Kuwae et al., 2008, 2012; Mathot et al., 2010). Biofilm, a thin film on the surface of intertidal estuarine mudflats is comprised of photosynthetic diatoms, cyanobacteria, invertebrates, and sediment bound in a polysaccharide matrix rich in carbohydrates and essential fatty acids (Cibic et al., 2007; Decho, 2000; Schnurr et al., 2019, 2020; Scholz & Liebezeit, 2013; Underwood, 2010). Fatty acids are components of lipid and serve as essential nutrients for shorebirds, providing fuel and improved flight performance for long‐distance migration (Guglielmo, 2010; Maillet & Weber, 2006, 2007; Quinn et al., 2017). Fatty‐acid content varies widely over the season due to three potential mechanisms: increase in diatom biomass, lipid accumulation response by microalgae, or changes in composition of the microbial community (Schnurr et al., 2019, 2020). As such, biofilm availability and factors affecting its ability to produce fatty acids at stopover sites may influence shorebird migration strategies.

On intertidal mudflats, biofilm abundance and fatty‐acid production are primarily influenced by seasonal fluctuations in environmental conditions. In the spring, increases in light and temperature trigger diatoms to reproduce and undergo rapid growth rates (Schwenk et al., 2013; Underwood & Smith, 1998). However, a wide variety of microalgae are also sensitive to changes in nutrient concentrations in the intertidal environment (Cibic et al., 2007; Decho, 2000; Underwood, 2010), so sudden changes in nutrient levels (e.g., nitrogen or silica), salinity, or other environmental stressors can trigger diatoms to accumulate lipid (Sharma et al., 2012). Freshwater input to estuarine mudflats increases during the spring freshet, a period of high discharge following snowmelt, which begins in spring and peaks in early summer. Freshwater released onto mudflats may induce sudden environmental changes, creating conditions favorable to some diatom species within the estuarine community, and triggering enhanced diatom lipid production, which increases the energy available for uptake by foraging shorebirds (Schnurr et al., 2019, 2020).

Estuaries are complex systems, constantly in flux, and particularly susceptible to changes in natural and anthropogenic processes including hydraulic control, channel modification, and climate change (Warwick et al., 1999). Changes to freshwater flow may have cascading effects on shorebird food availability and quality, which could influence shorebird site usage, body condition, and survival, and could ultimately contribute to population‐level changes (Baker et al., 2004). Despite growing evidence of links between freshwater flow, biofilm abundance and lipid production, and shorebird biofilm feeding on estuarine mudflats (Schnurr et al., 2019, 2020), there are no investigations into the co‐occurrence of shorebird presence and freshwater discharge at migratory stopover sites. While the dynamic nature of estuarine habitats must be accounted for to assess the overall function of the ecosystem (Warwick et al., 1999), trends in shorebird abundance and habitat use at some sites may be useful indicators of estuarine ecosystem health (Mathot et al., 2018).

The Fraser River Delta is a particularly important stopover site along the Pacific Flyway and is used by globally significant numbers of migrating Western Sandpiper (*Calidris mauri*), and Pacific Dunlin (*Calidris alpina pacifica*) (Drever et al., 2014; Iverson et al., 1996; Shepherd & Lank, 2004). In this study, we used count data collected from 1991 to 2019 during northward migration (April to May) at Roberts Bank, a large mudflat within the Fraser River estuary, British Columbia, Canada, to examine the relationships between abundances of Western Sandpiper and Pacific Dunlin, and local environmental conditions. While accounting for seasonal and annual trends in counts, we used a modeling approach to assess correlations between environmental parameters (Table 1) and shorebird abundance. We predicted strong north‐westerly winds, warm air temperatures, and low predation would correlate with high shorebird abundance and hypothesized that the influence of tidal amplitude, precipitation, and the influx of freshwater from the Fraser River during the spring freshet would affect habitat availability and biofilm function, and thus shorebird abundance.

**TABLE 1:** Rationale for covariate inclusion in the models of shorebird abundance at Roberts Bank, 1991 to 2019

Covariate	Rationale
Air temperature	Daily mean air temperature. Diatom growth is maximal under ideal (warm) temperature conditions. Spring shorebird migration tends to correlate with warm and rising temperatures (Richardson, 1978)
Precipitation	Daily total precipitation. Heavy precipitation could influence or dilute nutrient conditions on the mudflat and trigger enhanced fatty‐acid production in diatoms (Schnurr et al., 2020). Precipitation may also induce some invertebrates (e.g., clams) to migrate further down into the mudflat surface reducing the availability of infaunal prey (Jiménez et al., 2015)
Wind vectors: westerly and southerly (speed + direction)	Shorebirds may take advantage of assisting winds, preferring to depart stopovers during strong winds from a south‐eastern direction (Alerstam, 1979), or remain when strong headwinds occur
Solar radiation	Solar radiation provides light and heat for photosynthesis and can affect primary production of mudflat ecosystems. Diatom growth in intertidal biofilm communities is maximal under ideal light conditions, providing migrating birds with more resources (Schnurr et al., 2020; Sriharan et al., 1991)
Tidal amplitude	Total difference between daily maximum and minimum tidal height. Roberts Bank is an intertidal mudflat, and the foraging area available for shorebirds varies between neap and spring tides. Shorebirds may take advantage of periods of large tidal amplitude (spring tides) in which larger portions of the mudflat are exposed for longer periods of time. However, mudflats may also dry out and desiccate during such long periods of tidal exposure making foraging for biofilm and infaunal invertebrates more difficult (Jiménez et al., 2015)
Discharge	Discharge (flow) rate of Fraser River as observed at Hope Station. Rapid changes in salinity may result in changes in biofilm community at Roberts Bank, for example, diatoms in biofilm enhance lipid production during periods of osmotic stress, such as a large influx of freshwater leading to changes in salinity. (Schnurr et al., 2020)
Raptor abundance	Total count of Peregrine Falcons and Merlin observed during each survey. Data available only from 1997 onwards. Raptor abundance provides a measure of predation risk at a stopover site and may result in early shorebird departure or decreased length of stay (Lank et al., 2003; Ydenberg et al., 2004)

## 2 METHODS

### 2.1 Study site and species

Roberts Bank (49.058°N, 123.163°W) is a large clay‐rich mudflat (8 km^2^) situated south of the mouth of the Fraser River, British Columbia, Canada. Estuarine environments are rich in nutrients, and Roberts Bank receives freshwater inputs from the Fraser River as water is released into the delta and pulled south by the Strait of Georgia current (Sutton et al., 2013). Discharge from the Fraser River increases in March–May during the annual spring freshet and typically peaks in June of each year (Kostaschuk & Atwood, 1990).

Western Sandpiper and Pacific Dunlin are the most abundant shorebirds that feed on Roberts Bank during their northward migration (between mid‐April and mid‐May). Up to 42%–64% of the estimated Western Sandpiper population and 30%–50% of the Pacific Dunlin flyway population stopover to rest and refuel at Roberts Bank during northward migration (Drever et al., 2014). Pacific Dunlin and Western Sandpiper have specialized morphological features for foraging. Western Sandpiper tongues are coated in specialized spines that facilitate biofilm ingestion, which comprises 40%–70% of their daily energy intake, while Pacific Dunlin rely less on biofilm and use their longer bills to probe in the mud for benthic infaunal invertebrates (Elner et al., 2005; Jiménez et al., 2015). Some Pacific Dunlin also overwinter at Roberts Bank, and additional migrating Pacific Dunlin begin to arrive on the mudflat from March to April. Pacific Dunlin departure occurs first in mid‐April and overlaps with the arrival of migrating Western Sandpipers after the start of the Fraser River freshet. We studied the relationship between Western Sandpipers and Pacific Dunlin because these species have morphological adaptations which aid in biofilm feeding and, therefore, should be affected by changes in freshwater flow and associated changes to biofilm.

### 2.2 Shorebird surveys

Western Sandpiper and Pacific Dunlin surveys were conducted at Roberts Bank annually from 1991 to 2019, excluding 1993 and 1996 (Drever et al., 2014, Environment and Climate Change Canada, unpub. data). A total of 540 shorebird surveys were conducted over the study period (*n* = 7–28 surveys/year, median 23–surveys/year). Surveys were done at a consistent tide height (3.5 m; falling or rising depending on daily logistical considerations such as sunrise and sunset) to ensure equal mudflat area exposure, began on 15 April of each year, and continued until fewer than 1,000 birds were observed or until 15 May, whichever occurred first. During surveys, we counted shorebirds using the mudflat from a series of stops along an adjacent dike. To estimate counts of each species, we estimated the daily species ratio of Western Sandpiper to Pacific Dunlin in subsamples of individual flocks, and multiplied these ratios by total flock counts (for further survey methodology details refer to Drever et al., 2014).

### 2.3 Environmental correlates

We considered a suite of variables to test our hypotheses about correlations between abundances of Western Sandpiper and Pacific Dunlin, and environmental data collected at long‐term monitoring sites (Table 1). Mean daily temperature (°C), precipitation (mm), wind speed (km/h), and wind direction were recorded at the Vancouver International Airport (49°11′41.000″N, 123°11′02.000″W; ~15 km from site; ECCC 2019). We used trigonometry to convert the angle of wind direction and wind velocity into westerly (“*u*”) and southerly (“*v*”) wind vectors. A positive “*u*” vector indicates the strength of wind moving from the west while a positive “*v*” vector indicates the strength of wind moving from the south. Solar radiation (W/m^2^) was recorded at the University of British Columbia Totem Field climate station (49.2562°N, 123.2494°W; ~23 km from site; UBC, 2020). Maximum and minimum tidal heights were measured at Point Atkinson (49.3333°N, 123.2500°W; ~31 km from site), from which we calculated tidal amplitude as the difference between daily maximum and minimum tidal heights. Fraser River discharge (m^3^/s) data were collected at the Hope‐Fraser River hydrograph station (49°23′09″ N, 121°27′15″W; ECCC, 2019). The Hope‐Fraser River hydrograph station is situated ~150 km upstream from Roberts Bank; however, discharge is tightly correlated between stations along the southern reaches of the Fraser River (Thorne & Woo, 2011). Environment and Climate Change Canada notes that discharge data from 2017, 2018, and 2019 are still preliminary and subject to revision.

At Roberts Bank, the main predators of shorebirds are Peregrine Falcon (*Falco peregrinus*) and Merlin (*Falco columbarius*). To generate a measure of predation risk, the number of raptors present on the mudflat or involved directly in attacks on shorebirds during surveys was recorded during shorebird surveys at Roberts Bank since 1997.

### 2.4 Environmental change over the study period

We first analyzed temporal changes in environmental variables using generalized additive models (GAM; Wood, 2017), modeling each variable separately, to assess the degree to which local conditions might have shifted over time and over the migration period. For each environmental covariate, we characterized seasonal and annual variation by fitting a GAM with package *mgcv* in R (Wood, 2011). Each model included a smooth function for Day of Year (DOY) and year, with a maximum number of knots set to 5 to avoid overfitting the model (Wood, 2011).

### 2.5 Shorebird baseline trend models

Following Drever et al. (2014), we analyzed abundance measures of Western Sandpiper and Pacific Dunlin separately to allow for differing patterns between species. As a baseline trend model, we used a linear mixed‐effects modeling approach (package *lme4* in R; Bates et al., 2015), with log‐transformed counts as the response variables, fixed effects of Year, DOY, and DOY^2^, with DOY and DOY^2^ as random slopes, and Year as a random intercept, with a Gaussian error distribution (Supplementary Material). This model allowed the relationship between daily count and DOY to vary by year and assumed that yearly variation may be caused by environmental factors. Both DOY and Year were centered on their means and scaled by their standard deviations over the entire dataset.

### 2.6 Environmental correlates and annual population indices

To test our hypotheses and better understand how environmental variables affect daily shorebird abundances, we added all environmental variables to our baseline trend model and used a backward elimination process on this full model using the *step* function modified for package *lmerTest* in R (Kuznetsova et al., 2017; Supplementary Material). This stepwise algorithm iteratively drops variables from the full model and retains variables depending on *F*‐tests for fixed‐effect terms using Satterthwaite methods for denominator degrees of freedom (*α*‐level = 0.05). In addition to temporal variables from the baseline trend model, the full model included discharge, tidal amplitude, precipitation, daily temperature, solar radiation, and strength of westerly and southerly winds. All explanatory variables were centered on their means and scaled by their standard deviations over the entire dataset. Inference was based on the stepwise‐selected model, termed the “final model.” Examination of residual plots from final models revealed no major departures from assumptions of nonheteroscedasticity and normality of residuals. As an additional measure, we estimated effect sizes of retained variables by calculating the percent change in predicted values derived from the final model with the independent variable of interest set at its 0.10 and 0.90 percentiles, with remaining independent variables set at their median values.

To evaluate the role of raptors, we relied on data on predator counts during surveys, which were only available from 1997 onwards. Therefore, we added a parameter for total raptor abundance to the final model and tested its significance using a *t* test on the ratio of the parameter value to its *SE*.

To generate an index of the population for each year, we used the final model to predict counts for each year, estimated for the median day of year (DOY = 119, April 28 or 29, depending on leap year) for the whole dataset, and for the environmental correlates set at their observed median values for each year. This annual index was used primarily for illustrative purposes, and we based our inference of population trajectories on the parameter estimates from final trend models.

## 3 RESULTS

### 3.1 Environmental change over the study period

**FIGURE 1:**
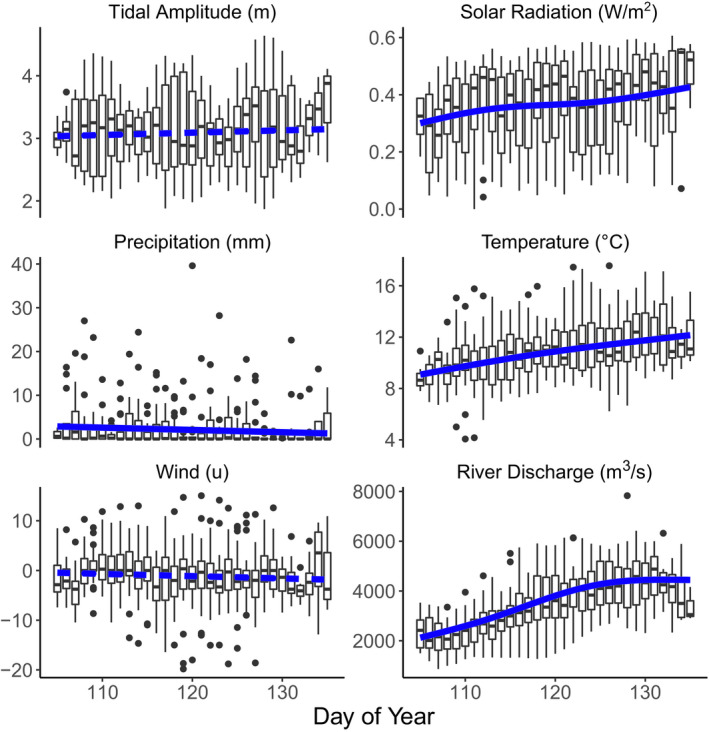
Seasonal changes in environmental covariates across the Fraser River estuary during the northward migration period for shorebirds (Day of Year (DOY) 105–135, ~April 15–May 15), 1991–2019. Blue lines depict predictions from General Additive Models (GAM), with solid lines indicating statistically (*F* > 3.9, *p* < 0.05) significant patterns over the season

**FIGURE 2:**
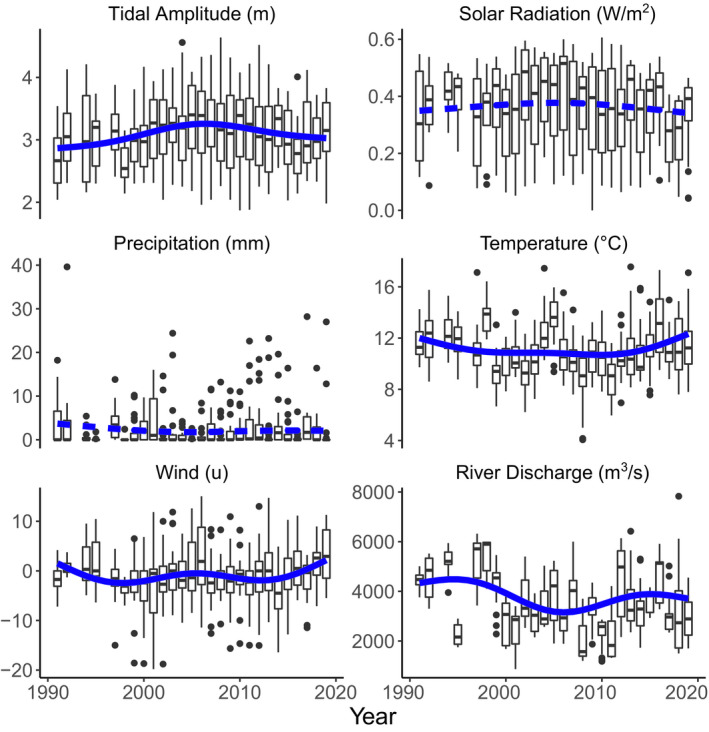
Annual changes in environmental covariates across the Fraser River estuary during the northward migration period for shorebirds, 1991–2019. Blue lines depict predictions from General Additive Models (GAM), with solid lines indicating statistically significant (*F* > 3.9, *p* < 0.05) patterns over the years

From 1991 to 2019, all environmental correlates showed significant seasonal variation or long‐term trends. Seasonal patterns indicated a strong tendency for increasing solar radiation, daily mean temperature, and river discharge rates from mid‐April to mid‐May, accompanied by decreasing daily precipitation (Figure 1). Tidal amplitude and both wind vectors (westerly and southerly) showed wide variability over the season, with no strong seasonal trend (Figure 1). Annual patterns also varied by environmental covariate, with solar radiation and precipitation showing no long‐term trend (Figure 2). River discharge rates, daily temperature, tidal amplitude, and wind vectors all had nonlinear fluctuations over the study period from 1991 to 2019, with a period in the mid‐2000s with lower average discharge rates, lower daily temperatures, and higher tidal amplitudes (Figure 2).

### 3.2 Baseline trends

The baseline trend model indicated strong seasonal variation in counts of both Western Sandpiper and Pacific Dunlin. Seasonal counts of both species varied quadratically with DOY. Pacific Dunlin indices peaked on ~16 April (DOY = 107) and declined thereafter, while Western Sandpiper indices typically increased from the start of the season and peaked on ~29 April (DOY = 120). The baseline trend model indicated a nonsignificant yearly decline in Western Sandpiper counts (*β*_yr_ = −0.134, *SE* = 0.083, *t* = −1.66, *p* = 0.11), and little change for Pacific Dunlin counts (*β*_yr_ = −0.014, *SE* = 0.076, *t* = −0.188, *p* = 0.85) from 1991 to 2019.

### 3.3 Shorebird abundance and environmental correlates

**FIGURE 3:**
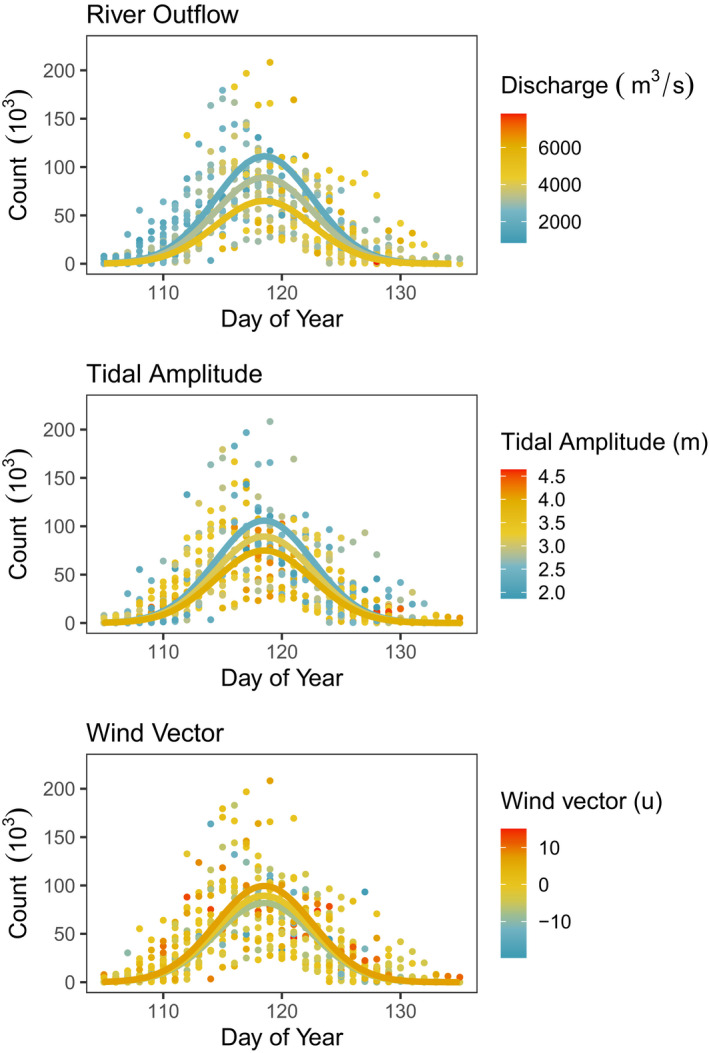
Relationships between Fraser River discharge, tidal amplitude, and westerly wind strength, with daily counts of Western Sandpiper at Roberts Bank, British Columbia. Lines indicate values predicted using the final model where other covariates were held at their medians, and variable of interest at 0.10, 0.50, and 0.90 percentiles. Not shown are counts above 225,000 birds (*n* = 12)

**FIGURE 4:**
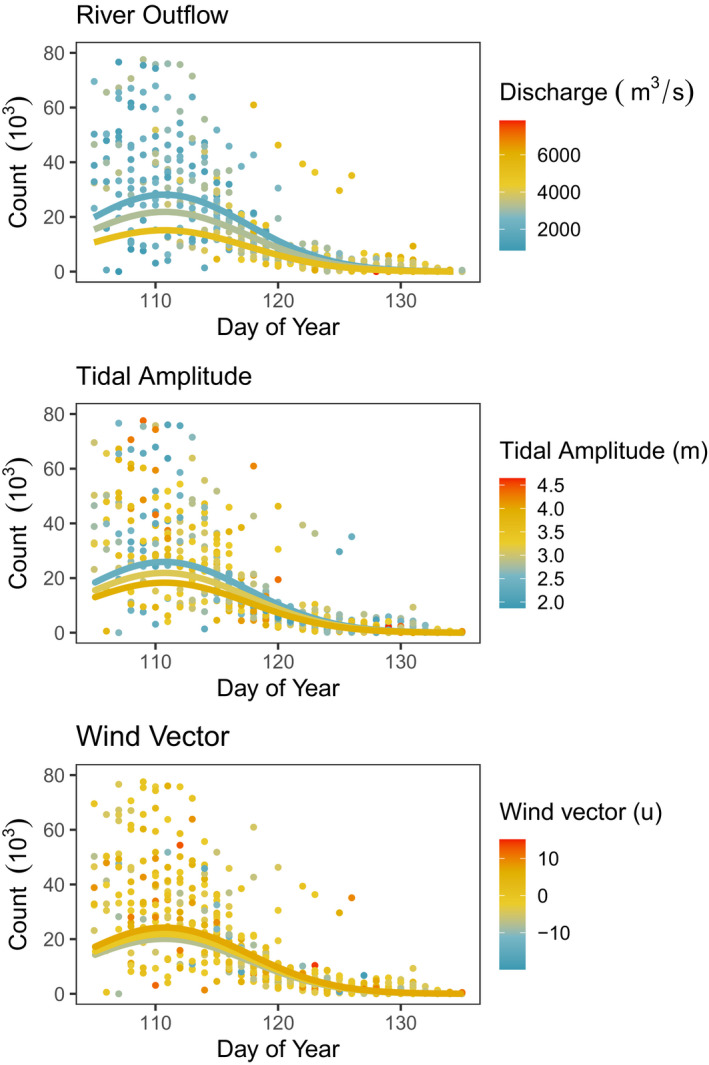
Relationships between Fraser River discharge, tidal amplitude, and westerly wind strength, with daily counts of Dunlin at Roberts Bank, British Columbia. Lines indicate values predicted using the final model where other covariates were held at their medians, and variable of interest at 0.10, 0.50, and 0.90 percentiles. Not shown are counts above 80,000 birds (*n* = 10)

In addition to the effects of Year, DOY, and DOY^2^, the final models for both Western Sandpiper and Pacific Dunlin included effects of river discharge, tidal amplitude, and westerly wind strength (Table 2; Figure 3). Shorebird abundances of both species were negatively influenced by river discharge and tidal amplitude (Figure 4), where fewer birds were seen during periods of high river discharge rates and wide tidal amplitude, and positively correlated with westerly wind strength such that more birds were observed at Roberts Bank on days with strong winds blowing from the west (Table 2; Figure 3). The parameter estimates and our measures of effect size indicated discharge had the strongest effect on shorebird counts, followed by tidal amplitude and westerly wind strength (Table 2). For Western Sandpiper, the final model also indicated that after accounting for these environmental variables, there was a significant long‐term decline in abundance (*β*_yr_ = −0.195, *SE* = 0.092, *t* = −2.13, *p* = 0.04; Table 2). However, for Pacific Dunlin we found a strong seasonal effect with DOY and DOY^2^ as significant predictors, but Year was not significant, indicating no long‐term changes in counts of Pacific Dunlin (*β*_yr_ = −0.092, *SE* = 0.083, *t* = −1.11, *p* = 0.27; Table 2). Comparison of models with and without predator measures using the reduced dataset from 1997 onwards indicated a marginally significant positive association between abundance of raptors and counts of Western Sandpiper (total raptor count: *β*_raptors_ = 0.075, *SE* = 0.044, *t* = 1.70, *p* = 0.09) and counts of Pacific Dunlin (total raptor count: *β*_raptors_ = 0.050, *SE* = 0.032, *t* = 1.53, *p* = 0.12).

**TABLE 2:** Parameter estimates for models identifying temporal trends and effects of environmental variables on counts of Western Sandpiper and Dunlin at Roberts Bank on the Fraser River Delta, British Columbia, 1991 to 2019

Variable	Estimate	*SE*	*t*‐value	*p*‐value	Effect size (%)
Western Sandpiper					
Fixed effects					
Intercept	11.363	0.117	97.22	<0.001	
Year	−0.195	0.092	−2.13	0.033	
DOY	−0.182	0.142	−1.29	0.198	
DOY^2^	−1.684	0.130	−12.96	<0.001	
Discharge	−0.200	0.090	−2.23	0.026	−71.2
Tidal Amplitude	−0.130	0.037	−3.53	<0.001	−41.3
Westerly wind	0.076	0.035	2.20	0.028	17.7
Random effects					
Year	0.546				
DOY	0.611				
DOY^2^	0.637				
Residual	0.718				
Dunlin					
Fixed effects					
Intercept	9.240	0.117	79.25	<0.001	
Year	−0.093	0.083	−1.11	0.266	
DOY	−1.314	0.152	−8.65	<0.001	
DOY^2^	−0.602	0.116	−5.19	<0.001	
Discharge	−0.231	0.087	−2.66	0.008	−86.0
Tidal Amplitude	−0.130	0.036	−3.59	<0.001	−41.5
Westerly wind	0.073	0.034	2.14	0.033	17.0
Random effects					
Year	0.547				
DOY	0.680				
DOY^2^	0.561				
Residual	0.708				

Note

Counts are ln‐transformed, and all independent variables were centered on their mean and scaled on their standard deviations. Effect size for environmental covariates calculated as the percent difference in predicted count between focal variable set at 0.90 and 0.10 percentiles.

### 3.4 Annual shorebird abundance indices

**FIGURE 5:**
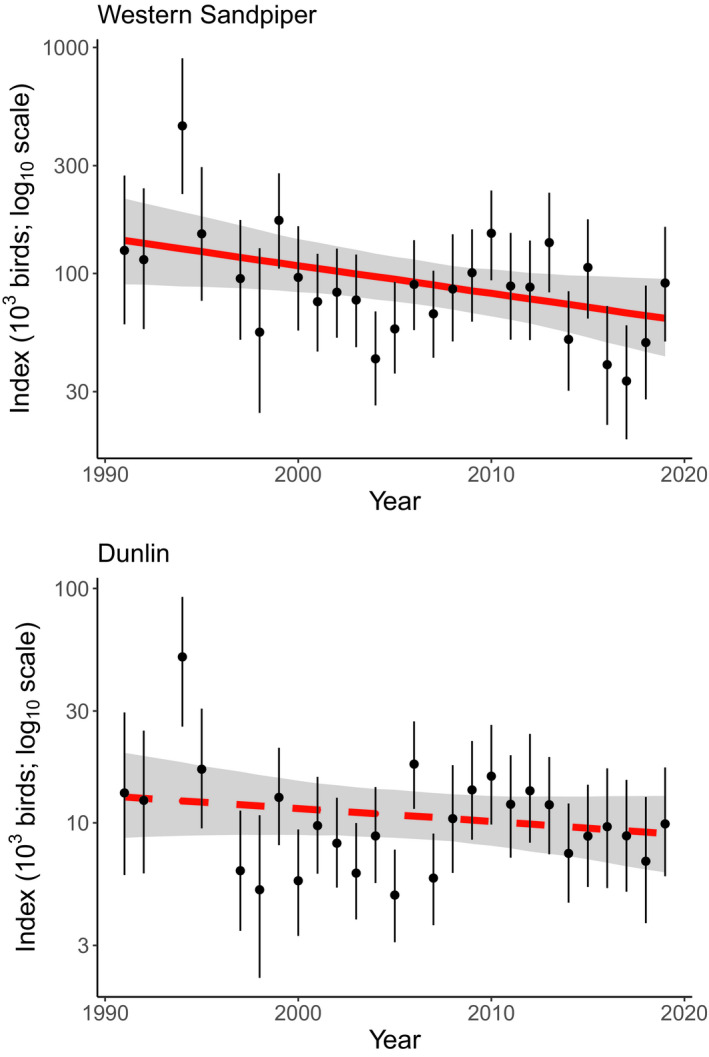
Trends in Western Sandpiper and Dunlin counts conducted at Roberts Bank between 1991 and 2019. Values represent population indices (with 95% confidence intervals) calculated as predicted values for each year from final models for each species (Table 2), with independent variables held at median values

The annual abundance indices for Western Sandpiper, derived from predicted values from the final model, varied widely over the study period (Figure 5), with highest values occurring in 1994 and lowest in 2017. These indices of Western Sandpiper showed a decline of 53.6% (−2.0% per annum) over the entire study period, and 24.0% over the last 10 years from 2009 to 2019 (−0.8% per annum). Similarly, annual indices for Pacific Dunlin varied widely from 1991 to 2019, with highest values in 1994 and lowest in 2005 (Figure 5). These indices showed a decline of −30.5% over the study period, but the trend model (Table 2) indicates this trend was not statistically significant.

## 4 DISCUSSION

Using a monitoring dataset from a 29‐year period from 1991 to 2019, we updated baseline trend estimates generated by Drever et al. (2014) for Western Sandpiper and Pacific Dunlin on the Pacific Flyway and analyzed correlations with environmental variables at Roberts Bank. Our results suggest a long‐term decline in Western Sandpipers, while Pacific Dunlin population indices have remained stable, and shorebird abundance at this site is correlated with westerly winds, tidal amplitude, and river discharge.

### 4.1 Baseline trends and annual indices

The negative trends in Western Sandpiper counts at Roberts Bank are consistent with negative trends in counts observed over much of the species’ North American wintering range (Meehan et al., 2018), supporting the contention that counts at Roberts Bank reflect the species’ status over a large fraction of its range. Similarly, our result of no long‐term trends in Pacific Dunlin counts are consistent with previous findings for the west coast of North America (Xu et al., 2015). The long‐term decline in Western Sandpiper abundances that we observed underscores the need to understand full lifecycle demography of this species, supports general trends in Arctic‐breeding shorebird populations (Smith et al., 2020), and suggests further conservation actions are needed to preserve this abundant species (Hope et al., 2019).

### 4.2 River dscharge

Timing of the Fraser River freshet is determined by the rate and quantity of snowmelt, compounded by spring precipitation and temperature within the Fraser River drainage basin (Kang et al., 2016). The lower numbers of shorebirds observed during days and years of higher discharge from the Fraser River likely reflects the effects of this discharge on food availability at Roberts Bank. To our knowledge, this study is the first to document a correlation with spring runoff and shorebird abundance at an estuarine stopover. Given the importance of intertidal biofilm in diets of shorebirds at Roberts Bank (Kuwae et al., 2008; Mathot et al., 2010; Jardine et al., 2015) and that microphytobenthos in biofilm can account for up to 50% of the primary production of estuarine ecosystems (Haro et al., 2020; Underwood & Kromkamp, 1999), it is possible that river discharge affects the quality of nutrients available on the mudflats where birds are foraging. Fatty acids in lipid provide essential nutrients to fuel long‐distance migration (Guglielmo, 2010), and the benthic diatoms in intertidal biofilm are a rich source of fatty acids (Scholz & Liebezeit, 2013). Diatoms at Roberts Bank are hypothesized to accumulate lipid when they experience fluctuations in salinity (Schnurr et al., 2020), as can occur during the early periods of Fraser River freshet in spring. Alternatively, the freshet may mark the seasonal successional change in the community composition of the microphytobenthos or its grazers, which would result in changes in the availability of fatty acids and other essential nutrients (Passarelli et al., 2015; Sahan et al., 2007). Despite their different dietary preferences, we found similar results for the impact of freshwater on Western Sandpiper and Pacific Dunlin, which could reflect the extent that freshwater flow influences the entire benthic community and not only diatoms and invertebrates. This correlational study cannot disentangle the underlying mechanisms surrounding growth and productivity in intertidal biofilm, but highlights the complexity of estuarine systems where the influence of freshwater incursion on shorebird abundance requires further study.

### 4.3 Tidal amplitude

Western Sandpiper and Pacific Dunlin abundances were negatively correlated with tidal amplitude, consistent with previous studies linking shorebird abundance with tidal amplitude (Fonseca et al., 2017; Granadeiro et al., 2006; Nehls & Tiedemann, 1993), and indicating that fewer birds were observed during spring tides (wide tidal amplitude). Tides in this system are semi‐diurnal (Thomson, 1981), and tidal amplitude is negatively correlated with the daily number of hours that the productive upper intertidal area remains exposed (*r* = −0.71; Environment and Climate Change unpub.). Therefore, we suggest that tidal amplitude would be closely related to the time and total area available for foraging by shorebirds (Calle et al., 2016; Granadeiro et al., 2006) and that more birds would be displaced to forage elsewhere during spring tides than during neap tides.

### 4.4 Wind

During spring migration, Western Sandpipers travel along the Pacific Flyway on a northwest axis from their nonbreeding grounds along the coast of South and Central America, Mexico, and California to breeding grounds in western Alaska. The higher abundance of shorebirds during periods of strong westerly winds could result from birds choosing to stay on the mudflat when winds are unfavorable for departure to the northwest (Alerstam & Lindström, 1990; Butler et al., 1997). At stopover sites, wind speed and direction affect bird departure, arrival and length of stay (Alerstam, 1979; Mitchell et al., 2015). Tail winds provide an advantage to migrating birds by reducing energy expenditure, which may increase flight speeds (Anderson et al., 2019; Shamoun‐Baranes et al., 2017), and headwinds or crosswinds can delay migration (Alerstam & Lindström, 1990). Similar relationships were found for shorebirds that delayed migration during unfavorable winds at stopover sites in the Yellow Sea and southern Scandiniavia (Grönroos et al., 2012; Ma et al., 2011). Our results indicate wind assistance is a factor, but that local conditions have a stronger influence than wind variables.

### 4.5 Complexity in estuarine ecosystems

Estuaries provide important ecosystem services including carbon storage, wave attenuation, sediment stability, coastline erosion prevention, and habitat for wildlife. Rich intertidal foraging habitat along coastlines has been an important feature in shorebird evolution of long‐distance migration in shorebirds (Butler et al., 2001). Worldwide, shorebird distribution is positively correlated with coastal primary productivity and shorebird stopover sites are selected based, in large part, on food abundance and availability (Butler et al., 2001). The discovery that biofilm is an important dietary component for Western Sandpiper (Elner et al., 2005; Kuwae et al., 2008), and a high‐energy food source for at least 21 additional species of shorebirds, including sandpipers, shanks, and plovers (Kuwae et al., 2012), has precipitated the need for a greater ecosystem‐level understanding of mudflats and estuaries (Mathot et al., 2018), as biofilm also provides food for benthic invertebrates that are in turn consumed by shorebirds (Cheverie et al., 2014; Hamilton et al., 2006). Our finding that shorebird abundance in the Fraser River Delta was affected by both marine and riverine processes highlights the complexity of estuaries, wherein the Fraser River interacts with the mudflat system at Roberts Bank resulting in larger ecological effects on shorebird migration. This complexity includes the possibility of feedback mechanisms in which faecal droppings from shorebirds, which add dissolved nutrients to the mudflat and are correlated with shorebird abundance (Canham, 2020), can stimulate growth of benthic diatoms (Jauffrais et al., 2015). Thus, research on the nutritional ecology of biofilm, invertebrates, and shorebirds, coupled with studies on how changing conditions in spring affect available nutrients, is needed to better understand the importance of estuarine environments to shorebird abundance during the critical northward migration period. We also acknowledge that our dataset is limited to daily counts of shorebirds and that information is lacking on stopover decisions or length of stay, an important indicator of stopover site quality (as addressed further in Drever et al., 2014). With the addition of a coordinated automated radio telemetry system (e.g., Motus, Taylor et al., 2017) across the Pacific Flyway, we believe tracking data will help to further elucidate shorebird stopover‐selection behavior in the Fraser River estuary and the results of this study.

### 4.6 Climate change implications

Many of the environmental parameters we examined are, or will be, influenced by climate change. Current climate models predict that mean surface temperature and mean precipitation will increase in mid‐latitude wet regions, and extreme precipitation events are likely to become more intense and more frequent (IPCC, 2014). Higher mean air temperatures may decrease snowpack in winter and result in earlier snowmelt, which will affect the size and timing of the Fraser River freshet (Shrestha et al., 2012). Given the uncertainty surrounding the factors affecting biofilm and its importance as a fuel source for migrating shorebirds, the influence of climate change on the spring freshet could have adverse effects on biofilm and thus food quantity and quality at this stopover site. If the advancement of freshet results in greater discharge rates during the critical northward migration period (15 April–15 May), then further declines in shorebird numbers may be expected.

## 5 CONCLUSIONS

The spring freshet is a transformative event on the Fraser River estuary, as freshwater and nutrients empty into the Strait of Georgia, coinciding with shorebird northward migration stopover. The Fraser River estuary provides a critical link in the Pacific Flyway stopover chain for shorebird populations migrating northward, and the river interacts with the Roberts Bank mudflat habitat in complex ways. However, much of the shoreline surrounding the Fraser River Estuary, including portions of Roberts Bank, is industrialized or has been converted to residential properties, and further coastal development is likely. Given the significant effects of wind strength and direction, tidal amplitude, and river discharge on shorebird abundance that we observed at Roberts Bank, the impacts of climate change on the size and timing of spring freshet could put an additional strain on habitat for global shorebird populations. As threats to coastal estuarine ecosystems increase with climate change and further development, protection of stopover site habitats will be a crucial component for shorebird conservation.

## CONFLICT OF INTEREST

The authors declare no conflicts of interest.

## AUTHOR CONTRIBUTIONS

**Rachel Canham:** Conceptualization (supporting); data curation (lead); formal analysis (supporting); investigation (equal); project administration (lead); writing – original draft (lead); writing – review and editing (lead). **Scott A. Flemming:** Formal analysis (equal); investigation (equal); methodology (equal); validation (equal); visualization (equal); writing – review and editing (equal). **David D. Hope:** Formal analysis (supporting); investigation (supporting); methodology (supporting); validation (equal); writing – review and editing (supporting). **Mark C. Drever:** Conceptualization (lead); data curation (supporting); formal analysis (lead); methodology (supporting); writing – original draft (equal); writing – review and editing (equal).

### Open research badges

This article has earned an Open Data Badge for making publicly available the digitally‐shareable data necessary to reproduce the reported results. The data is available at https://doi.org/10.5061/dryad.ghx3ffbmj.

## Supporting information

Supplementary Material

## Data Availability

Shorebird count data and environmental correlate data from this study are available from the open data portal Dryad (https://doi.org/10.5061/dryad.ghx3ffbmj).
